# Acquired Perforating Dermatosis in a Patient on Peritoneal Dialysis: A Case Report and Review of the Literature

**DOI:** 10.1155/2018/5953069

**Published:** 2018-01-22

**Authors:** Talha H. Imam, Hassan Patail, Nabeela Khan, Phillip T. Hsu, David S. Cassarino

**Affiliations:** ^1^Department of Nephrology, Southern California Permanente Medical Group, Fontana Medical Center, 9961 Sierra Ave, Fontana, CA 92335, USA; ^2^Pulmonary and Critical Care, Stony Brook University Hospital, Stony Brook, NY 11794, USA; ^3^Memorial Sloan Kettering Cancer Center, New York, NY 10065, USA; ^4^Department of Dermatology, Southern California Permanente Medical Group, Fontana Medical Center, Fontana, CA 92335, USA; ^5^Department of Pathology, Southern California Permanente Medical Group, Los Angeles Medical Center, Los Angeles, CA 90027, USA

## Abstract

Acquired perforating dermatosis (APD) is a debilitating and itchy skin disease. Its diagnosis is based on biopsy and the treatment is not very clear. It is not well established as to how wide spread it is in patients on peritoneal dialysis (PD) and its implications in this population have not been well studied. Here we present a case of APD that developed in a patient on PD. Its pathology and treatment options are reviewed. More studies are needed to assess the prevalence of APD in PD population.

## 1. Introduction

Perforating dermatoses are a diverse group of diseases, where there is transepidermal elimination of material from the dermis. These have been reported in hemodialysis patients but not much in peritoneal dialysis patients. We report a patient on peritoneal dialysis (PD) who developed acquired perforating dermatosis with debilitating itching.

## 2. Case Report

A 60-year-old obese patient with diabetes mellitus (DM) developed end-stage renal disease (ESRD) and subsequently was initiated on PD in October 2012. The etiology of his ESRD was DM. Prior to the start of his PD, his HbA1C ranged from 6.5% to 13.5%. His peritoneal equilibrium test (PET) showed his membrane transport characteristic to be of high average. His prescription included five nightly cycles, with two-liter fill volume of dextrose solutions for nine hours. He had his last fill of two liters of icodextrin for ten hours and another mid-day exchange with two liters of dextrose. Over the next couple of years, he did well on PD until April 2015 when he developed culture negative peritonitis. In May 2015, he developed Candida peritonitis (he also had groin candidiasis) and his PD catheter was promptly removed. He was then switched to hemodialysis (HD) after insertion of a HD catheter in his neck. In June 2015, he was admitted with septic shock and expired.

During his time on dialysis all his *KT*/*V* were greater than 2.0. During the year prior to his death, his albumin ranged from 2.7 to 3.6 mg/dl, phosphorus 3.8 to 6 mg/dl, calcium uncorrected 7 to 8.5 mg/dl, and parathyroid hormone immunochemiluminescence 127 to 993 pg/ml. After initiation of his PD, his HbA1C ranged from 6.5% to 8.6%. His medications included insulin, nortriptyline, sevelamer carbonate, cinacalcet, lisinopril, montelukast, multivitamins, and epoetin alpha.

**Figure 1:**
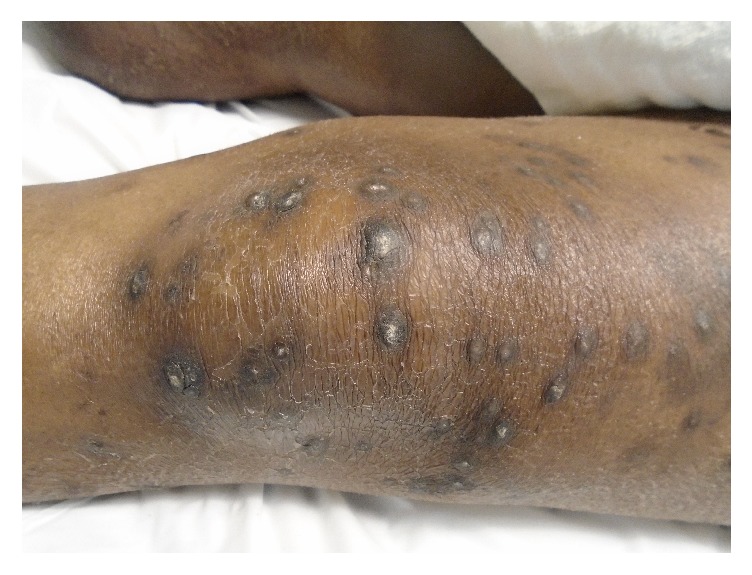
Dorsum of left knee.

**Figure 2:**
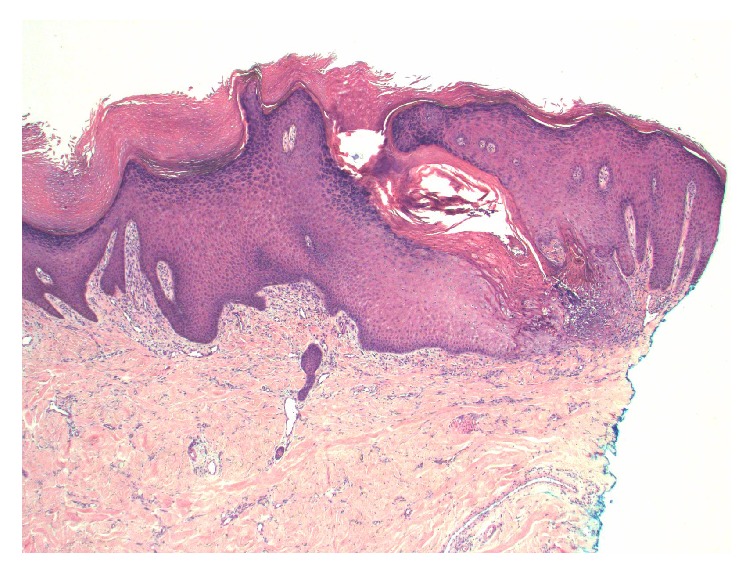
Low magnification view showing irregular epidermal acanthosis, with a prominent transepidermal defect containing cellular debris (hematoxylin and eosin stain, original magnification ×40).

**Figure 3:**
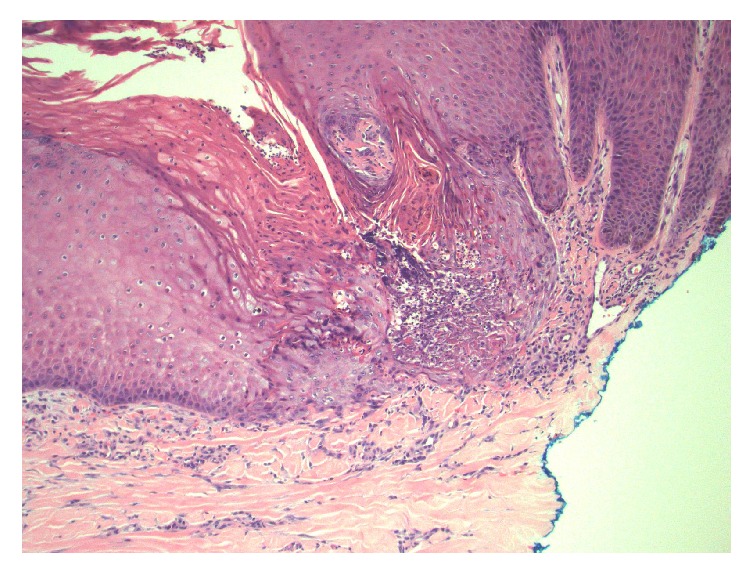
Higher magnification showing the area of defect containing neutrophils, parakeratosis, and degenerating keratinocytes (hematoxylin and eosin stain, original magnification ×200).

**Figure 4:**
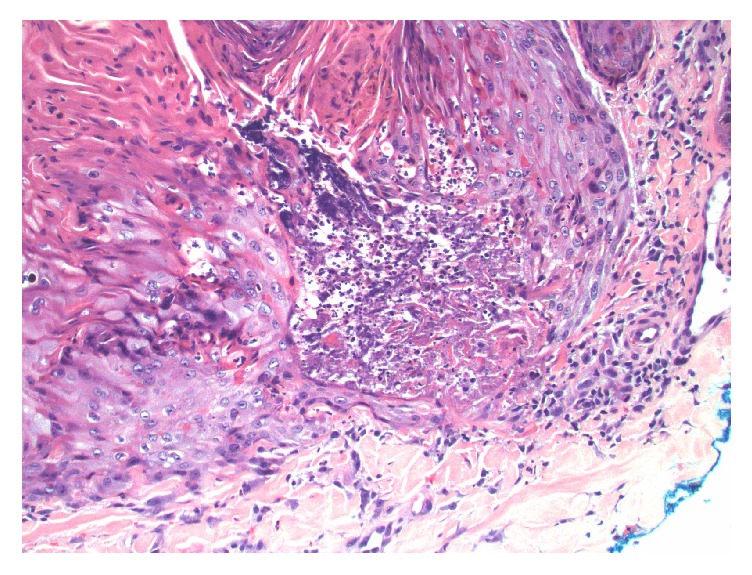
High magnification of the base of the defect showing perforation through the epidermis, and communicating with the superficial dermal collagen (hematoxylin and eosin stain, original magnification ×400).

He had started seeing a dermatologist in May 2014 for newly developed pruritic hyperpigmented papules and nodules around 0.5 mm to 1 cm in size. These were mostly on his face, arms, and legs (Figure 1). He was very disturbed due to itching. He started taking nortriptyline and applying gold bond lotion, but without much improvement. A punch biopsy of one of the skin lesions revealed changes consistent with an acquired perforating dermatosis (APD) (Figures 2, 3, and 4). Conservative treatment with triamcinolone 0.1% cream did not improve the intense pruritis. Tazarotene 0.1% gel was started but was too irritating for the patient. He was switched subsequently to tretinoin 0.1% cream in hopes of better tolerability. There was a discussion with the patient about possibly starting oral isotretinoin or narrow band UVB therapy, but the patient passed away from sepsis.

## 3. Discussion

The perforating dermatoses are a diverse group of diseases that include four types: elastosis perforans serpiginosum, Kyrle disease, perforating folliculitis, and reactive perforating collagenosis (RPC). Rapini et al. first introduced the term APD in 1989 [1]. Seen mostly in middle-aged adults with a mean age of 48, APD is a rare disorder associated with systemic diseases, mainly chronic kidney disease (CKD) and DM [2]. Though APD can occur prior to initiating dialysis, it more commonly occurs after a patient is placed on dialysis [3]. It occurs at a rate of 4.5–11% in the dialysis population, and most of the literature shows an association with HD rather than PD [4]. APD can also occur at sites of trauma or after exposure to certain medications, including TNF-alpha inhibitors, bevacizumab, sirolimus, and indinavir.

APD presents as a rash with umbilicated papules and a central keratotic cap, commonly associated with pruritis [2]. Lesions most often present on the extensor surfaces of the lower extremities, but APD can also exhibit on the trunk, scalp, or any area that the patient may scratch due to pruritis.

Although the pathogenesis of APD remains unclear, there are many hypotheses. Patients will often exhibit the Koebner phenomenon, where lesions arise at the sites of trauma exhibited by scratching [5]. One theory postulated consists of self-induced trauma to the skin resulting in damage to the epidermis or dermal collagen which eventually causes APD [1, 2, 6]. Another theory is an alteration of collagen or elastic fibers due to metabolic disturbances or micro-deposition of substances such as calcium salts [4]. Finally, dermal vasculopathy associated with diabetes leading to necrosis is thought to possibly play a role.

Skin biopsy is diagnostic for APD, and histopathology reveals findings of any of the four perforating dermatoses, and often more than one pattern is observed in a single patient [1, 2]. Therefore, many prefer to lump all these diagnoses into one umbrella term of APD. Histology often reveals epidermal invagination often involving a dilated hair follicle with a keratotic plug consisting of keratin, collagen, or elastic fibers associated with neutrophils. The clue to diagnosis is that a central keratotic core should be seen overlying a focus of epidermal perforation.

Individual lesions can resolve spontaneously, so if there is mild involvement, avoiding trauma and scratching by trimming fingernails, wearing gloves, application of menthol solution, or behavior modification can be sufficient. For limited but significant involvement, treatments include topical retinoids, such as tretinoin or tazarotene, topical or intralesional corticosteroids, emollients, imiquimod cream, laser ablation, and cryotherapy. For more extensive involvement antihistamines, oral retinoids (isotretinoin, acitretin), methotrexate, allopurinol, or phototherapy [7, 8] (narrowband UV-B, broad band UV-B, or psoralen UV-A) may be appropriate. Some patients have improvement after renal transplantation or anecdotally after change in HD tubing or equipment. In the clinical setting, however, the most common treatment modalities used are avoidance of scratching, menthol lotions, oral antihistamines, topical steroids, topical retinoids, narrow band UV-B, or doxycycline. While oral retinoids and systemic steroids may be used, the coexisting conditions often seen in these patients, such as renal disease, often make it challenging to use these safely.

Very limited information is available about APD in the PD population [4, 5, 9, 10]. There have been known cases of APD in the PD population, as seen in the literature from Saray, and Morton et al., but there have been no direct studies comparing HD and PD populations. What we know so far is that APD has a more causal relationship to kidney disease than to DM. But we do not know if dialysis, more importantly the type of dialysis, changes the prevalence, outcome, or sequela of this disease. In the study of 22 patients by Saray et al., two patients were diagnosed with APD once PD was started [2]. A case reported by González-Lara et al. revealed a patient who was diagnosed with APD after PD, with improvement after being switched to HD [8]. These findings suggest that there may be a causal relationship between APD and PD itself, although it may not be the same mechanism as its relationship to HD. The itching and pain associated with it can further add to depression, which can potentially lead to more infections in PD. More epidemiological studies are needed to evaluate the prevalence of APD in patients on PD and the risk factors associated with APD in the PD population.
